# Ancestral proteins trace the emergence of substrate specificity and oligomerization within bacterial DEDDy dinucleases

**DOI:** 10.1126/sciadv.aef1581

**Published:** 2026-08-12

**Authors:** Sofia Mortensen, Andrew A. Burnim, Keith Dufault-Thompson, Alexandra E. Lipka, Xiaofang Jiang, Holger Sondermann

**Affiliations:** ^1^ CSSB Centre for Structural Systems Biology, Deutsches Elektronen-Synchrotron DESY, Notkestr. 85, 22607 Hamburg, Germany.; ^2^ National Center for Biotechnology Information, National Library of Medicine, National Institutes of Health, 8600 Rockville Pike, Bethesda, MD 20894, USA.; ^3^ Christian-Albrechts-Universität, 24118 Kiel, Germany.

## Abstract

Nucleases are crucial for various bacterial processes, including genome maintenance and host defense. Deoxydinucleases (diDNases), a class of Gram-positive bacteria–specific nucleases associated with mobile genetic elements, are homologous to nanoRNase C (NrnC) in Gram-negative bacteria but exhibit notable differences: diDNases form dimers and cleave DNA dinucleotides, whereas NrnC forms octamers that process both RNA and DNA dinucleotides. The mechanism by which substrate specificity emerged, and whether it is linked to oligomerization, remained unknown. Here, we reconstructed a common ancestor of diDNases and NrnC orthologs that forms a dimer with intermediate preference for DNA. Structures of ancestral and extant dinucleases reveal gradual changes in conformation that gave rise to substrate preference, oligomeric state, and catalytic efficiency. These findings highlight how subtle, concerted structural modifications enable large-scale changes in molecular assembly and functional specialization, harnessing a conserved protein fold. DNA dinucleotide preference in the early ancestor and preservation of DNase activity in all extant enzymes strongly argue for a biological function of DNA dinucleotides.

## INTRODUCTION

Nuclease activity is fundamental to many bacterial processes, including DNA replication, RNA processing and turnover, and defense against intruding genetic elements. Although most nucleases facilitate catalysis via well-established strict geometries, catalytic residues, and ion requirements, their substrate preferences are much less predictable (*1*). Some nucleases, which are thought to be redundant owing to their high sequence and structural similarities, show unexpected differences in substrate specificities when analyzed biochemically, as exemplified by recent studies on nanoRNases. For example, DHH-DHHA1 family nanoRNase A (NrnA) and nanoRNase B (NrnB) comprise very similar domains but display distinct preferences with regard to substrate length and directionality (*2*, *3*): NrnA is a 5′ exonuclease that cleaves single-stranded nucleic acids of two, three, and four nucleotides; in contrast, NrnB is a processive 3′ exonuclease that has a C-terminal, positively charged extension that allows rapid processing of both short and long substrates. Similarly, oligoribonuclease (Orn) and ribonuclease T (RNase T) adopt a common DnaQ-like fold with a catalytic DEDDh motif (*4*, *5*). While RNase T processes longer nucleic acid substrates, Orn has a peculiar preference for 5′-phosphorylated dinucleotides.

We recently found another notable difference in substrate preference among homologous nucleases, particularly DEDDy-type dinucleases. One group of these enzymes comprises housekeeping nanoRNase C (NrnC) orthologs found in Alphaproteobacteria, Cyanobacteria, and Spirochaetia (*6*, *7*), whereas the other, termed deoxydinucleases (diDNases), is preferentially encoded by mobile genetic elements (MGEs) of Actinomycetes and Clostridia (*8*). Both diDNases and NrnC orthologs adopt a conserved DnaQ fold that harbors the DEDDy motif at the respective active sites and supports strict two-Mg^2+^ ion catalysis to cleave phosphodiester bonds. However, at the primary sequence level, the two enzyme subfamilies have distinctive signatures within their catalytic exonuclease I (EXO I), EXO II, and EXO III motifs (*9*). In addition, there are notable differences between diDNases and NrnC orthologs regarding their substrate preferences and physiological roles. As their name indicates, diDNases are highly specific toward 5′-phosphorylated, single-stranded DNA of two nucleotides in length, deoxydinucleotides, whereas NrnC enzymes cleave both DNA and RNA dinucleotides with equal efficiency (*8*). Although NrnC, as well as the functionally analogous Orn, has been implicated in the essential process of catalyzing the final degradation step of RNA and RNA-based second messengers, the relevance of their activity on DNA remains unclear (*4*, *6*, *7*, *10*, *11*). In contrast, on the basis of their genomic neighborhoods, diDNases are predicted to play a role in DNA mobility or phage defense (*8*). Overall, the high sequence and structural similarities between NrnC orthologs and diDNases are in notable contrast to their differential activities and physiological roles.

Another difference between NrnC and diDNase proteins pertains to their quaternary structures. Both enzymes dimerize using similar interfaces; however, NrnC orthologs assemble further into octamers comprising four dimeric subunits, whereas diDNases do not show signs of higher oligomerization beyond a constitutive dimeric form (*8*). The importance of the higher-order oligomeric arrangement of NrnC remains unclear.

A structural comparison of the enzyme-substrate complexes of diDNase and NrnC revealed that the functional differences between the nucleases could not be explained simply by invoking localized single amino acid residue substitutions; rather, the differences appeared to rely on a sum of seemingly disjointed conformational alterations in these enzymes (*8*). This observation is in agreement with the fact that evolution of elaborate biochemical features in proteins such as substrate specificity, allostery, or high-order assemblies generally requires substitution of many amino acid residues. Studying the evolutionary steps in those cases via a horizontal approach of mutating key residues while keeping the extant protein sequence background is often unproductive and rather requires a vertical approach of bioinformatically resurrecting probable ancient protein sequences and analyzing their biochemical properties (*12*–*14*). Ancestral sequence reconstruction was used in several cases where stepwise accumulation of many substitutions including changes in both the key and supporting residues led to protein functional diversification. For example, diversification of mineralocorticoid and glucocorticoid receptors to recognize either aldosterone or cortisol, respectively, using the same overall protein fold was traced to epistasis, where the effect of key mutations depended on the presence of permissive sequence changes (*15*). In another study, it was necessary to perform ancestral sequence reconstruction to trace incremental small transitions in the green fluorescent protein superfamily that led to the emergence of red-colored proteins (*16*). Here, we used bioinformatics to resurrect protein ancestors of extant diDNases and NrnC orthologs to unravel the emergence of apparent variations in biochemical properties within highly similar folds. We present a functional analysis of the reconstructed ancestral proteins, revealing that the evolution of diDNases and NrnC orthologs likely involved an ancestor with intermediate properties that bifurcated toward a higher or lower preference for DNA dinucleotides. We also determined crystal structures of ancestral and extant dinucleases, which allowed us to identify the structural alterations in the DnaQ fold that led to the emergence of the specific activities of extant nucleases. These studies also revealed that NrnC octamerization is correlated with increased overall enzymatic activity and a shift in substrate preference toward both DNA and RNA dinucleotides.

## RESULTS

### Resurrection of diDNase/NrnC ancestral proteins

diDNases and NrnC orthologs share considerable sequence similarity and adopt a common fold; however, they have notable distinctions in substrate preference and quaternary structure (*8*), which cannot be readily explained by localized sequence differences between the two DEDDy protein subfamilies. To develop a fundamental understanding of how the differential properties of these enzymes evolved in a shared fold, we chose a bioinformatics approach relying on ancestor reconstruction.

**Fig. 1.**
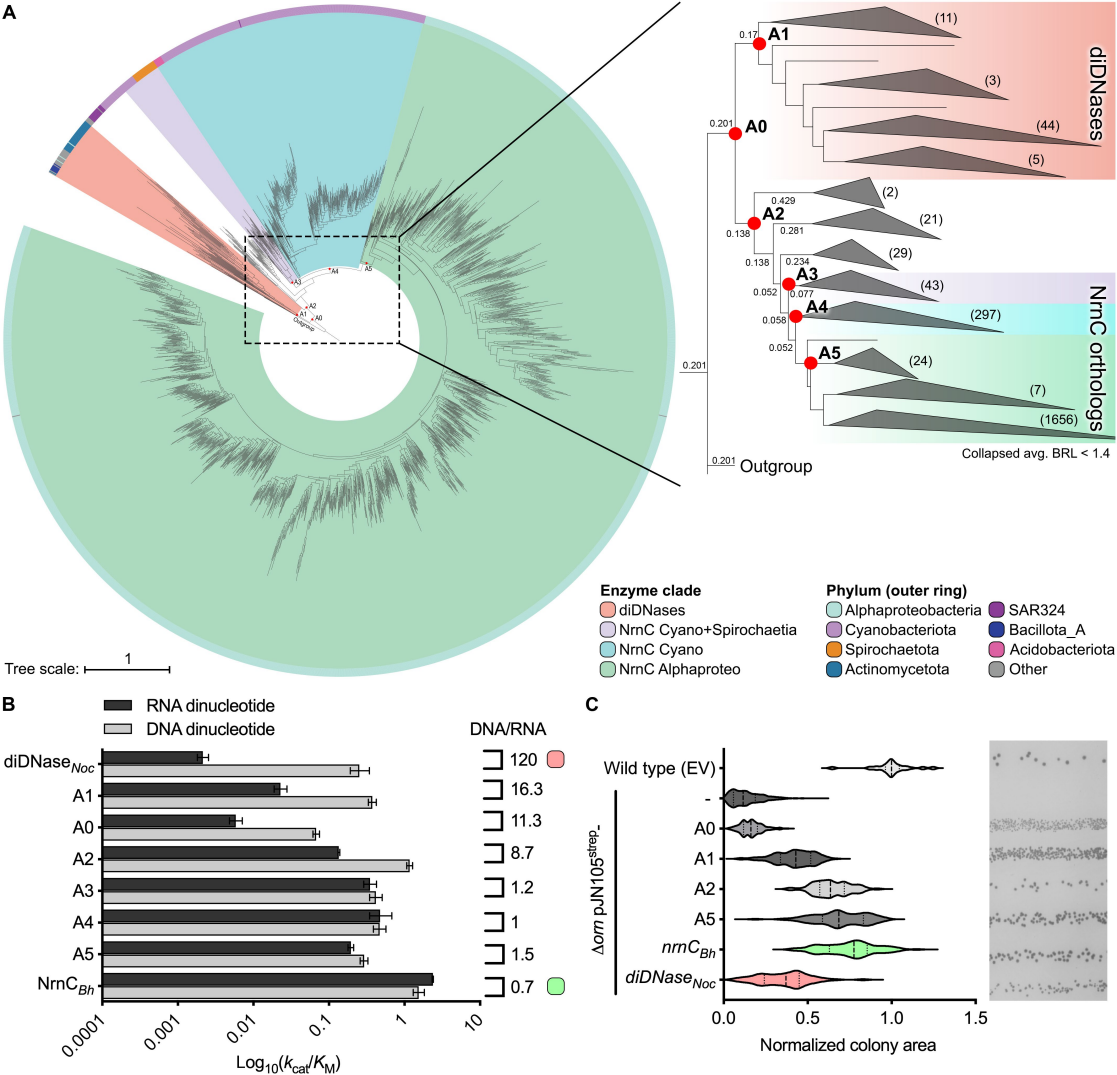
Ancestral protein reconstruction of diDNases and NrnC orthologs. (**A**) Phylogenetic tree with diDNase and NrnC clades, manually rooted using homologous outgroup sequences. Taxonomic assignments were mapped onto the outer ring. Branch lengths between important nodes are shown in the right panel with close-up view of the ancestral nodes between extant diDNase and NrnC clades. Right panel: Leaves of the tree with average branch length <1.4 were collapsed, and the number of leaves in each collapsed clade is stated in parentheses. The ancestral nodes chosen for characterization are marked with red dots. BRL, branch length. (**B**) Catalytic efficiency of ancestral proteins toward DNA and RNA dinucleotides, dp(2AP)G and p(2AP)G, respectively. The values of *k*_cat_/*K*_M_ (×10^6^ M^−1^ s^−1^) are plotted on a log_10_ scale. The error bars show the 95% confidence interval of the Michaelis-Menten model fitting. The efficiency values of diDNase from *Nocardioides* (diDNase*_Noc_*) and NrnC from *Bartonella* (NrnC*_Bh_*) are plotted for reference (*8*). Relative DNA/RNA specificities are listed. (**C**) Complementation of the small-colony phenotype of *P. aeruginosa* Δ*orn* using the indicated ancestral proteins and controls of NrnC*_Bh_* and diDNase*_Noc_*. Violin plots show colony sizes normalized to the average size of wild-type *P. aeruginosa* colonies from the same plate. A representative image of an assay plate used for scoring is shown in the right panel.

We constructed a comprehensive phylogenetic tree of NrnC orthologs, diDNases, and closely related homologous proteins and mapped the proteins with known substrate preferences on the tree on the basis of previous reports (Fig. 1A) (*7*, *8*). Phylogenetic reconstruction indicated that diDNase and NrnC sequences form two deeply separated clades within the dinuclease family, with the NrnC clade then further splitting into distinct subclades associated with the Cyanobacteria, Spirochaetia, and Alphaproteobacteria phyla (Fig. 1A). The bootstrap values for the split between the diDNases and NrnC groups were well supported (bootstrap >90). However, the support values for the splits between the NrnC clades were relatively low, making it difficult to determine the branching order within the NrnC subfamily. We then used ancestral sequence reconstruction to predict the likely ancestral sequences of the enzymes at key branching points in the tree. We identified five key nodes on the basis of the tree topology that represented common ancestors and divergence points between the clades of interest (Fig. 1A, right panel). We designated the sequence of the common diDNase/NrnC ancestor A0, the ancestor of the diDNase clade A1, the ancestor of the entire NrnC clade A2, and the ancestors of the subclades of NrnC A3, A4, and A5. Sequence alignment of the ancestors showed that the characteristic DEDDy dinuclease signatures at the EXO I, EXO II, and EXO III motifs and at the C terminus emerged independently and in parallel within NrnC and diDNase lineages later than the A0, A1, and A2 branching points (fig. S1A).

To understand the evolution of the substrate specificity of diDNases, which have a strong preference for DNA, and NrnC proteins, which do not discriminate between RNA and DNA dinucleotides as substrates, we recombinantly expressed the resurrected ancestral sequences, purified the proteins, and measured their enzymatic activities toward RNA and DNA dinucleotides containing the fluorescent purine analog 2-aminopurine (2AP) (Fig. 1B; fig. S1, B and C; and tables S1 to S3). We used three types of base compositions as the substrate series, namely purine-purine (p[2AP]G or dp[2AP]G), purine-pyrimidine (p[2AP]C or dp[2AP]C), and pyrimidine-purine (pC[2AP] or dpC[2AP]). All the ancestors were enzymatically active and exhibited robust nuclease activity. However, both common diDNase/NrnC ancestor A0 and diDNase ancestor A1 displayed somewhat lower levels of activity toward all substrates than extant NrnC orthologs and diDNases (*8*). Ancestor A0 had the lowest overall level of activity among all measured ancestral and extant proteins with 66 to 442 times lower activity for RNA and 7 to 23 times lower activity for DNA. In the case of ancestor A1, the activity compared to that of extant enzymes was 16 to 104 times and 1 to 4 times lower for RNA and DNA, respectively (table S1). These data indicate that the dinuclease activity of the proteins became more robust after the emergence of the common ancestors A0, A1, and A2. Nevertheless, while ancestors A0, A1, and A2 cleaved both DNA and RNA nucleotides, they demonstrated higher catalytic efficiency toward DNA than RNA dinucleotides (Fig. 1B, fig. S1B, and table S1). Comparative analysis of extant enzymes and reconstructed ancestors is consistent with increasing specialization toward DNA substrates in the diDNase clade and reduced discrimination between DNA and RNA substrates in the NrnC clade. In the NrnC lineage, the preference for DNA substrates was completely lost after the ancestor A2, leading to extant NrnC proteins, with ancestors A3 to A5 displaying roughly equal activity on RNA and DNA dinucleotides (Fig. 1B; fig. S1, B and C; and tables S1 to S3). In the lineage leading to diDNases, a strong preference for DNA emerged after the ancestor A1. Given that ancestor A1 already had an activity level toward DNA substrates that was comparable to that of extant diDNases, the DNA preference of diDNases originated from evolutionary pressure on A1 to lose RNase activity. The same pattern of gradual changes in nuclease activities and RNA/DNA preference is seen for all tested substrates independent of base composition (fig. S1, B and C, and tables S1 to S3). In summary, we showed that reconstructed ancestral enzymes exhibit intermediate substrate preferences relative to extant diDNase and NrnC proteins, supporting a model in which present-day specificities arose through divergence from a less specialized ancestral state in two directions: toward a stronger DNA preference in the diDNase clade and a lack of preference in the NrnC clade.

To examine whether the in vitro enzyme kinetics correlated with the dinuclease activities of the ancestors in vivo, we tested the ability of ancestors A0, A1, A2, and A5 to complement the small-colony phenotype of *Pseudomonas aeruginosa* Δ*orn* (Fig. 1C and fig. S1D). Orn has comparable activity toward RNA and DNA dinucleotides, and a complete rescue of the phenotype of the *orn* deletion strain depends on this feature (*8*). Hence, the level of small-colony phenotype complementation indicates whether a protein acts as an RNA/DNA nondiscriminating dinuclease or an enzyme with DNA preference, for example, a diDNase, which would result in an incomplete phenotype rescue (*4*, *7*, *8*). Ancestor A0 provided almost no complementation, consistent with its low overall nuclease activity and slight preference for DNA, as observed in vitro. Ancestor A1 could only rescue to an average level of 0.5 relative to the colony size of wild-type *P. aeruginosa*. A similar complementation potential was observed for an extant diDNase (Fig. 1C) (*8*). Ancestors A2 and A5 showed complementation levels on par with those of nondiscriminating NrnC orthologs. Together, these results corroborate the substrate preferences of the ancestral proteins observed in vitro.

### Structural basis for the emergence of substrate preferences in diDNases and NrnC orthologs

In extant diDNases and NrnC orthologs, substrate preference has been shown to correlate with a combination of large-scale structural features affecting dimer conformation as well as more localized features, mainly implicating amino acid residues at positions 80, 87, and 205 [per *Bartonella henselae* NrnC (NrnC*_Bh_*) numbering]. However, it remained unclear to what extent the large-scale differences contribute to substrate restrictions. In addition, the nature of residues 80, 87, and 205 alone could not fully explain the apparent substrate preferences, given that mutations swapping those residues between NrnC and diDNase did not affect specificity or led to impaired overall enzyme activity (*8*). Therefore, we used the resurrected proteins to investigate whether the emergence of large-scale structural features between ancestors and extant enzymes, particularly in the dimeric protein core, tracks the divergence in the substrate preferences of these enzymes.

**Fig. 2.**
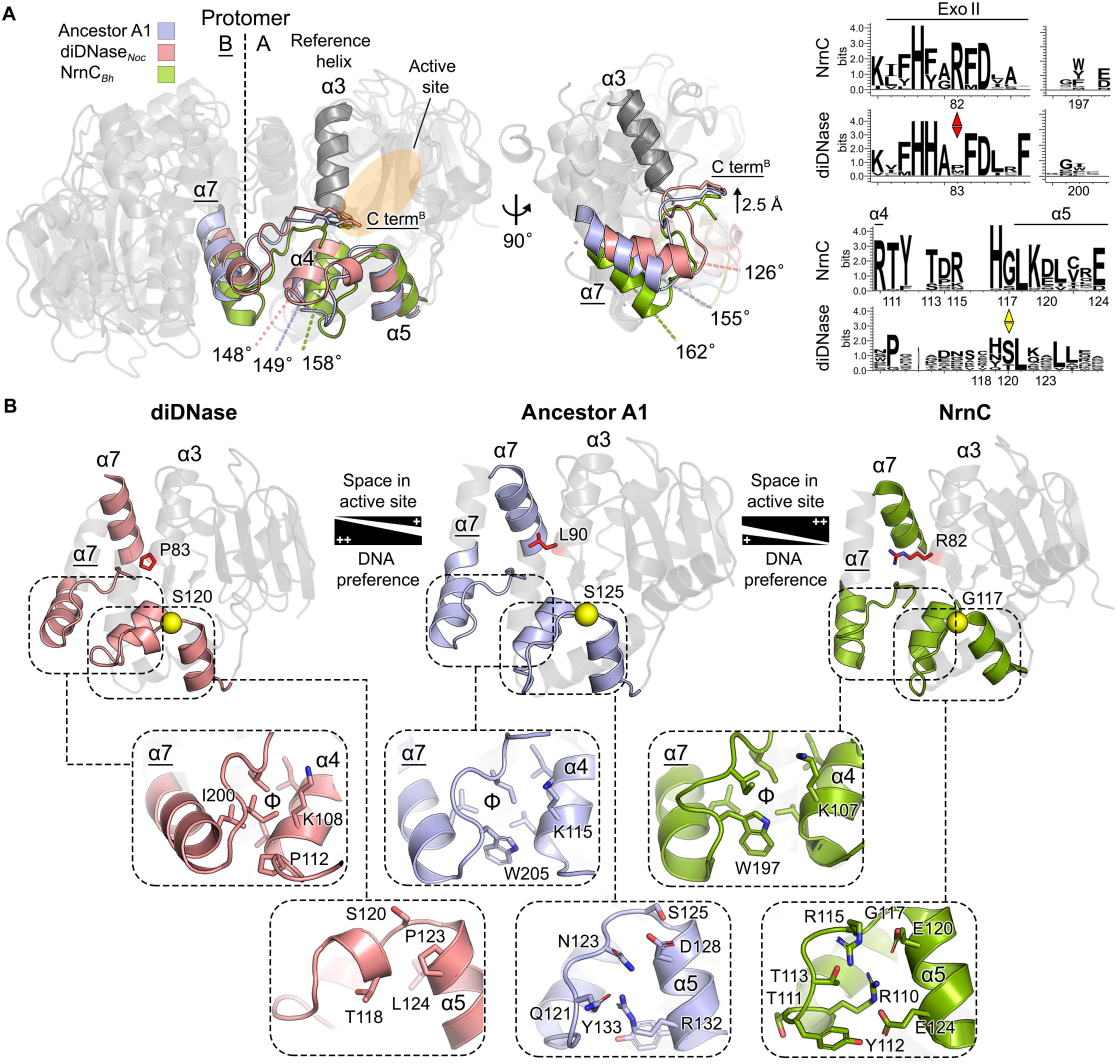
Structural basis for the evolution of substrate preferences in diDNases and NrnC orthologs. (**A**) Comparison of structures of extant enzymes, diDNase*_Noc_*-dpGG (PDB ID: 9F7G), NrnC*_Bh_*-pGG (PDB ID: 7MPL), and ancestor enzyme A1. The structures were aligned with the catalytic motifs EXO I and EXO II as references. The well-aligned helix α3 in protomer A was chosen as a reference to measure the angles between helix α3 and helices α4 and α7, which are implicated in large conformational changes leading to the differential ability of the active site to accept RNA as substrates. Right panel: Sequence logos of the regions identified by structural analysis as defining the amount of space in the active site and, hence, the preference for DNA. (**B**) Detailed views of the structural differences, which cause alteration in positions of helices α4 and α7, in extant diDNase, NrnC, and ancestral A1 protein. The hinge position occupied by either glycine (G) or serine (S) is shown as a yellow sphere, and the position of the arginine wedge (R82 in NrnC) is shown in the top panels as a side chain with red carbon atoms. The Greek letter phi marks a hydrophobic patch formed by helices α4 and α7 of an adjacent molecule.

To this end, we determined a crystal structure of an ancestor with an intermediate level of DNA preference and a high level of deoxyribonuclease (DNase) activity, ancestor A1, at a 1.45-Å resolution. Given that it has been shown previously that the presence of substrates in the active sites of NrnC and diDNase does not lead to any major conformational changes (*7*, *8*), we compared the protein entities of the structures of diDNase*_Noc_*-dpGG [Protein Data Bank (PDB) ID: 9F7G], NrnC*_Bh_*-pGG (PDB ID: 7MPL), and ancestor A1 by aligning them on the basis of the catalytic motifs EXO I and EXO II of their respective protomer A (Fig. 2A). Consistent with previous observations (*8*), the alignment showed differences in the position of the C terminus of protomer B in the active site of protomer A and, consequently, variations in space at the active site for a substrate in all three structures. In addition, the C terminus–proximal helix α4 of protomer A and helix α7 of protomer B are situated differently, which can be quantified by the angles between the well-aligned reference helix α3 and helix α4 of protomer A and between helix α3 of protomer A and helix α7 of protomer B (Fig. 2A). The angle between helices α3 and α4 ranged from 157.6° in NrnC to 147.8° in diDNase; in ancestor A1, the corresponding angle was 149°. The angle between α3 and α7 ranged from 162.2° in NrnC to 126.4° in diDNase compared to the two helices forming a 155.1° angle in ancestor A1. In the diDNase structure, the C terminus, along with helices α4 and α7, comes closer to the reference helix α3 and the active site of protomer A than in NrnC. Similar to the angle measurements, these structural elements adopted intermediate positions in ancestor A1 (Fig. 2A). This result, together with our enzyme activity data, suggests that alterations within just 2.5 Å of how deep the C terminus of one protomer is placed into the active site of the other protomer in the dimeric unit were crucial in tuning the preference for DNA up or down during diDNase and NrnC evolution. These alterations were caused by changes in the conformation and relative positioning of helices α4 and α7.

To rationalize these changes, we examined the factors influencing the conformations of the helices and their interactions with other elements (Fig. 2B). The ability of helix α4 to form a wider angle with helix α3 in NrnC can be attributed to a conserved glycine, G117, which acts as a hinge between helices α4 and α5 (Fig. 2, A and B). This feature provides more flexibility than the serine residue at the same position in diDNase and ancestor A1. Another NrnC-specific element that wedges between helix α7 and the C terminus and prevents the C terminus from reaching deeper into the active site is the arginine residue R82 (Fig. 2B). Notably, R82 is highly conserved as part of the EXO II motif in NrnC (Fig. 2A). In ancestor A1 and diDNase*_Noc_*, the analogous positions are occupied by smaller and uncharged residues, leucine and proline, respectively. In addition, in NrnC and ancestor A1, unlike in diDNase, there are several polar interactions between helix α5 and the α4-α5 loop (Fig. 2B, bottom panel). The residues participating in these interactions are conserved in NrnC orthologs (Fig. 2A). The distance between helices α4 and α7 in both NrnC and ancestor A1 is slightly larger than that in diDNases, likely due to the presence of a conserved bulky tryptophan residue, W197 in NrnC and W205 in ancestor A1, as part of a hydrophobic interface between the helices (Fig. 2B, middle panel). Together, the ancestral structure A1 has a mixture of NrnC- and diDNase-specific elements, ultimately leading to an intermediate level of DNA preference.

### Coemergence of substrate promiscuity and octamer assembly in NrnC orthologs

**Fig. 3.**
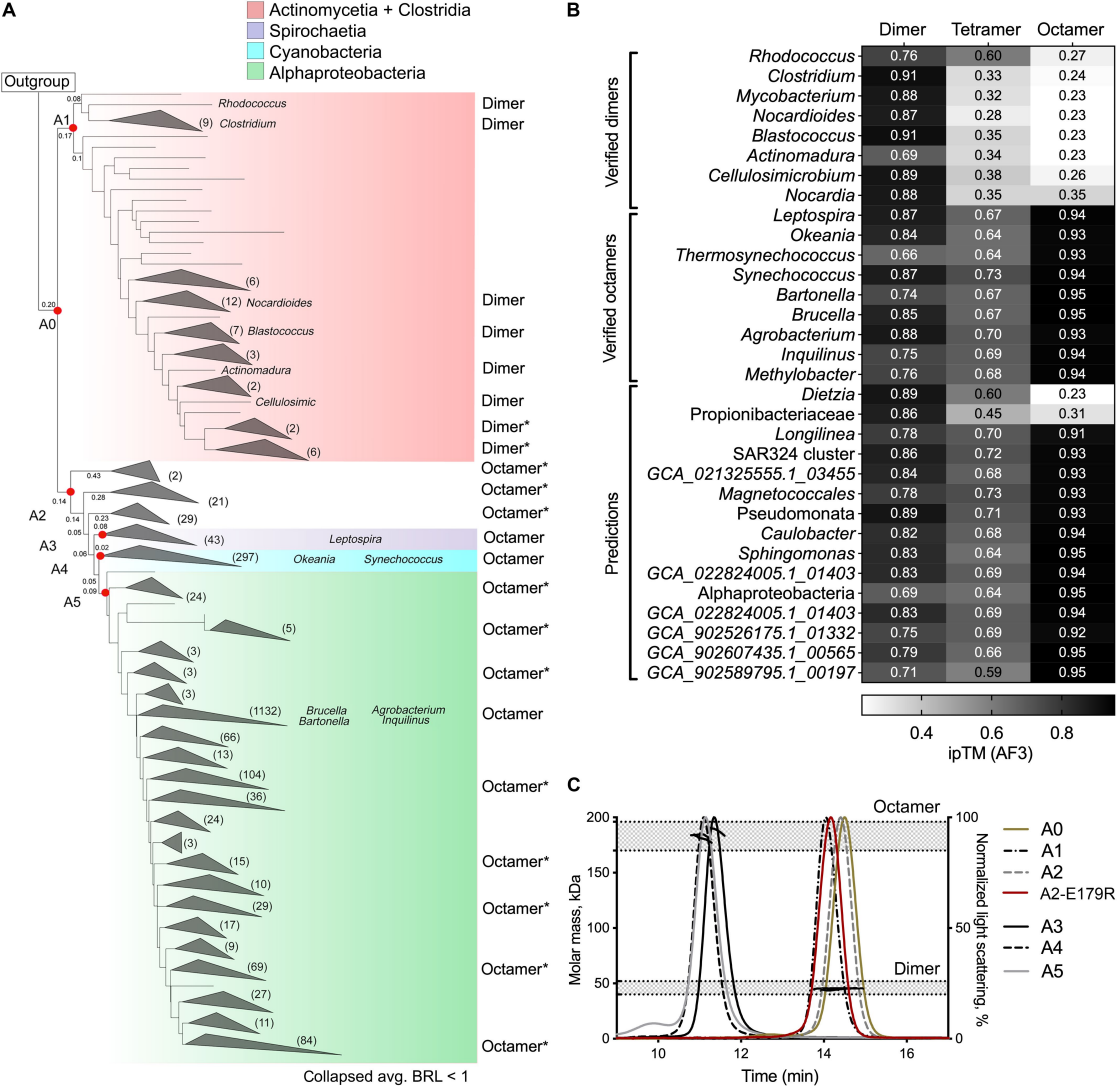
Emergence of octamerization in NrnC. (**A**) Mapping of the oligomeric state phenotype onto the phylogenetic tree and ancestral nodes. The branch lengths between the major nodes are shown. Leaves of the tree with average branch length <1.0 were collapsed, and the number of leaves in each collapsed clade is stated in parentheses. Ancestral nodes chosen for characterization are marked with a red dot. Oligomeric states determined experimentally or using AF3 predictions (marked with asterisks) are indicated. (**B**) AF3 was used to predict the oligomeric states of NrnC orthologs and diDNases. The ipTM scores of the dimeric, tetrameric, and octameric states of different proteins are plotted as a heatmap. The experimentally determined (verified) oligomeric states are indicated. (**C**) Oligomeric state of ancestral proteins determined by SEC-MALS. The normalized light scattering signal (chromatogram lines, right axis) and molar mass values (dots across peaks, left axis) were plotted against the SEC elution volume. The shaded areas show the theoretical molar mass ranges for dimeric and octameric assemblies on the basis of the primary sequence of the proteins.

In addition to distinct substrate preferences, NrnC orthologs and diDNases differ in their oligomeric states (*8*). Experimental oligomerization data for 12 extant enzymes have established that diDNases form stable dimers, whereas the NrnC orthologs analyzed so far exist as tetramers of analogous dimeric units, forming an overall octameric assembly (*7*, *8*, *17*). The previous analysis covered orthologs from *Rhodococcus*, *Nocardioides*, *Clostridium*, *Blastococcus*, *Cellulosimicrobium*, *Bartonella*, *Brucella*, *Agrobacterium*, *Inquilinus*, *Leptospira*, *Okeania*, and *Synechococcus*. This selection sparsely covered the phylogenetic tree. To further increase the confidence in an NrnC/diDNase classification of self-assembly forms, we determined the dominant oligomeric state in solution of additional NrnC and diDNase orthologs, particularly those from *Methylobacter*, *Thermosynechococcus*, *Nocardia*, *Actinomadura*, and *Mycobacterium*, using size-exclusion chromatography–coupled multiangle light scattering (SEC-MALS) (fig. S2, A and B). These orthologs followed the same characteristics as observed previously, with NrnC proteins forming octamers and diDNases forming dimers (Fig. 3A). To cover parts of the phylogenetic tree lacking experimental data, we used AlphaFold3 (AF3) to predict oligomeric states. We first assessed whether the experimentally determined quaternary structures were predicted with high confidence scores [i.e., interface-predicted template modeling (ipTM) scores]. The results established that the predictions matched the experimental SEC-MALS data (Fig. 3B). In particular, although all sequences were predicted to assemble into dimers, only the NrnC subgroup had intermediate or high confidence scores when modeled as tetramers or octamers, respectively. On the basis of these results, we applied this approach to additional orthologs from across the phylogenetic tree (Fig. 3, A and B). Plotting the quaternary structure predictions onto the tree confirmed a strict furcation of octameric NrnC proteins and dimeric diDNases (Fig. 3A).

Next, to determine the appearance of the octamerization propensity during NrnC evolution and functional specialization, we experimentally characterized the oligomeric states of the ancestors using SEC-MALS (Fig. 3C). Ancestors A3, A4, and A5 eluted as octamers. In contrast, ancestors A0, A1, and A2 formed stable dimers under identical conditions. Phylogenetic mapping of experimentally determined quaternary structures showed that the common diDNase/NrnC ancestor A0 was likely a dimer, and the diDNase lineage maintained this trait without developing a tendency to assemble into higher oligomeric structures (Fig. 3A). NrnC ancestors starting from A3 form octamers, suggesting that NrnC proteins acquired this trait after ancestor A2 and retained it throughout evolution. This observation indicates that the ability to form octamers occurred early in evolution in the transition between A2 and subsequently emerging orthologs.

**Fig. 4.**
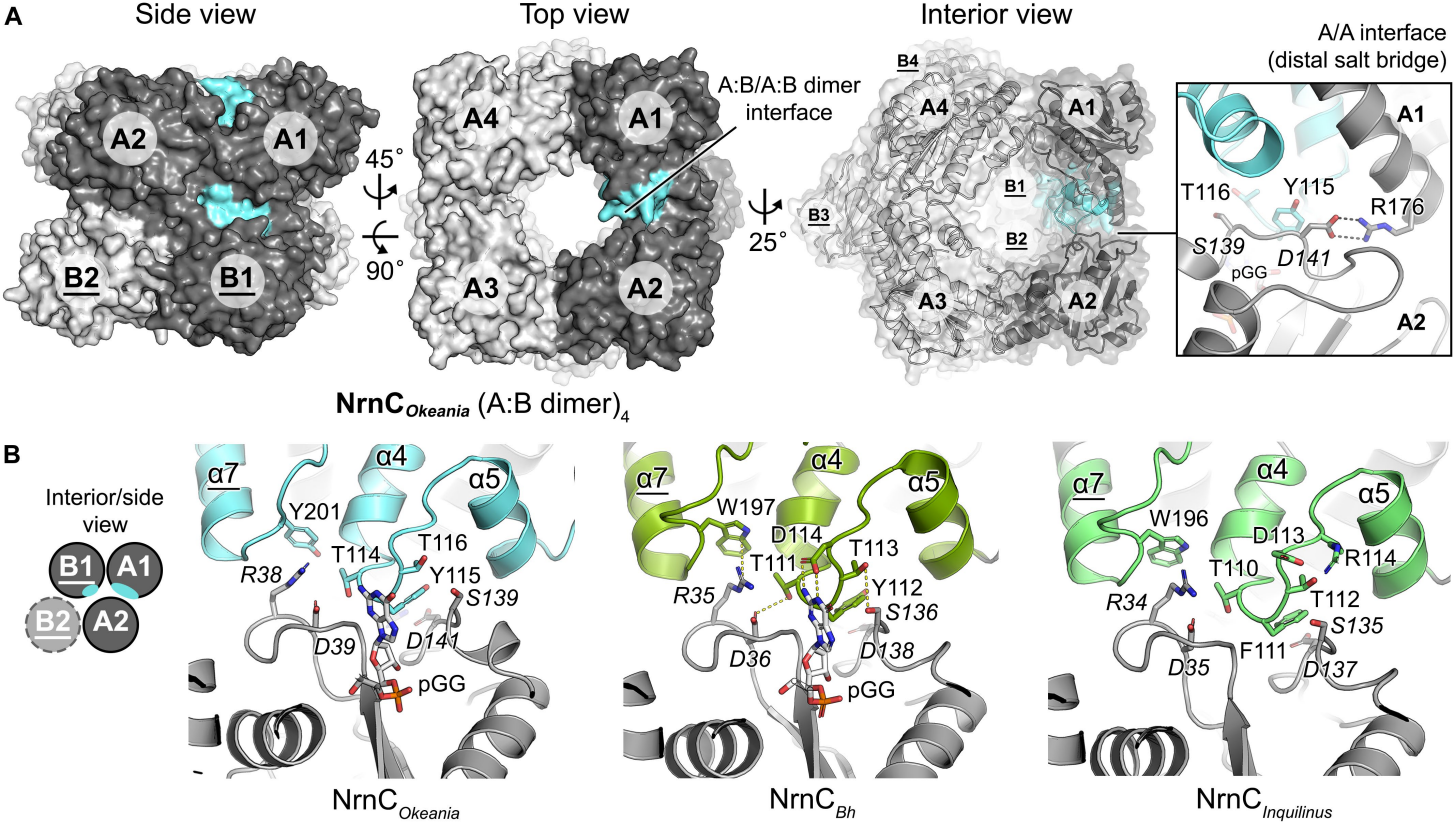
Conserved structural basis for octamerization in NrnC. (**A**) Structural analysis of the NrnC*_Okeania_* octamer. Four A/B homodimers tetramerize to form an octamer. As a result, A and B subunits form tetrameric rings. Top and side views are shown. The interfacial surface of dimer subunit is colored. The close-up view shows a conserved salt bridge that is part of the octamerization interface. (**B**) Structural comparison of the octamerization interfaces of *Bartonella*, *Okeania*, and *Inquilinus* NrnC. The main interacting residues are shown as sticks. Main secondary structure elements were labeled for orientation. Underlined labels refer to the secondary structure element of protomer B1; cursive labels refer to residues of protomer A2.

On the basis of our results, loss of DNA substrate preference and the emergence of octameric assemblies are both associated with the NrnC clade and appear to have arisen over the same broad evolutionary interval (Figs. 1 and 3). Next, we investigated the structural basis for the apparent coemergence of oligomer switching and substrate preferences. The analysis is based on the octamerization contacts between the dimeric subunits of the available structures of *Bartonella*, *Brucella*, and *Agrobacterium* NrnCs from the Alphaproteobacterial order Hyphomicrobiales (*7*, *17*). Moreover, we determined two additional crystal structures: (i) that of an additional Alphaproteobacterial NrnC ortholog from *Inquilinus* of the order Rhodospirillales and (ii) that of a Cyanobacterial NrnC ortholog from *Okeania* of the order Oscillatoriales (Fig. 4). The NrnC ortholog from *Inquilinus* crystallized in the apo state. NrnC*_Okeania_* crystals grew in the presence of dinucleotide pGG after bivalent cations were removed from the protein by chelation to prevent catalysis. From the 32 NrnC protomers (four octamers) in the asymmetric unit, three had additional density at their active sites accounting for pGG. For the other protomers, two guanosine 5′-monophosphate moieties were observed, probably because of trace amounts of bivalent cations that remained bound to the protein and supported catalysis during crystallization. The coordination of uncleaved pGG by NrnC*_Okeania_* is identical to that observed for Alphaproteobacterial NrnC orthologs.

Octamerization of NrnC*_Okeania_* and NrnC*_Inquilinus_* relies on conserved interfaces described previously for Alphaproteobacterial NrnC orthologs (Fig. 4) (*7*, *17*). The octamers assemble by ring formation of four homodimeric subunits, generating stacked upper and lower tetramer rings with a central tunnel (Fig. 4A). Ring closure involves several structural motifs. In the reference structure of NrnC*_Bh_*, the loop between helices α4 and α5 participates in interactions between adjacent subunits inside the rings via main chain interactions involving regions 29 to 36 and 135 to 140 as well as two hydrogen bonds, one between side chains of threonine T111 and aspartate D36 and the other between T113 and serine S136 (Fig. 4B, middle panel). In addition, residue W197 creates an inter-ring cation-π bond with R35 (Fig. 4B, middle panel). In NrnC*_Okeania_*, corresponding interactions involve residues T114 and T116 from the α4-α5 loop with side chains of D39 and S139 from the neighboring subunit as well as tyrosine Y201 and R38 (Fig. 4B, left panel). Both elements indicated by the structural analysis to stabilize octamers, the α4-α5 loop and an aromatic residue around position 200 (e.g., W197 of NrnC*_Bh_*), were also implicated in defining substrate promiscuity of NrnC (Fig. 2B). Only one intra-ring contact, a salt bridge between the side chains of R176 and D141 in NrnC*_Okeania_* (R172 and D138 in NrnC*_Bh_*), is established by residues not implicated in determining substrate specificity (Fig. 4A, right panel). All these specific interactions are not only conserved between NrnC*_Okeania_* and NrnC*_Bh_* but also in NrnC*_Inquilinus_* (Fig. 4B, right panel).

The interactions described above are repeated within the octameric assembly, contributing to the overall stability of the quaternary structure in crystals and solution (fig. S2B) (*7*, *8*, *17*). As discussed above, the α4-α5 loop in NrnC adopts a particular conformation that also contributes to creating more space in the active site of the protein, which allows RNA dinucleotides to bind (Fig. 2, A and B). Together, substrate specificity and octamerization rely on overlapping structural elements (Figs. 2 and 4). In contrast, the conformation of the α4-α5 loop and the position of residue corresponding to W197 of NrnC*_Bh_* (Y201 in NrnC*_Okeania_*) in diDNases are not compatible with NrnC-like intersubunit contacts, as both elements are too far apart to engage in such interactions (fig. S2C).

The emergence of octamerization can be traced to the same point in ancestry when one considers the structural elements that are involved in octameric interfaces and the primary sequence of extant and ancestral enzymes (Fig. 3A and fig. S1A). Ancestor A2 has most of the structural elements that hold subunits together in octameric NrnC assemblies, namely motifs resembling ^35^RDR^37^, ^110^RTYTDRHG^117^(α4-α5 loop), and W197 (following NrnC*_Bh_* numbering). However, this ancestor lacks an arginine residue corresponding to NrnC*_Bh_* R172 to form the R172-D138 salt bridge (fig. S1A). We investigated whether the mutation of the glutamate residue at the correspoding position to arginine, E179R, in ancestor A2 would be sufficient to switch the protein to an octameric state in solution. SEC-MALS data showed that the A2-E179R mutant remained dimeric (Fig. 3C), suggesting that other features important for octamerization are still lacking in ancestor A2, possibly involving the signature in the EXO II motif and/or the length of the C terminus (fig. S1A).

In addition to intersubunit interactions, the α4-α5 loop in NrnC proteins participates in the coordination of the 3′ base of the dinucleotide substrate of the neighboring subunit, either via main-chain interactions as seen in the structure of NrnC*_Okeania_*-pGG or via both main-chain and side-chain (D114) interactions as observed in NrnC*_Bh_*-pGG (Fig. 4B). Next, we explored the relevance of this coordination, and octamerization in general, for NrnC function.

### Role of octamerization for NrnC enzymatic activity

**Fig. 5.**
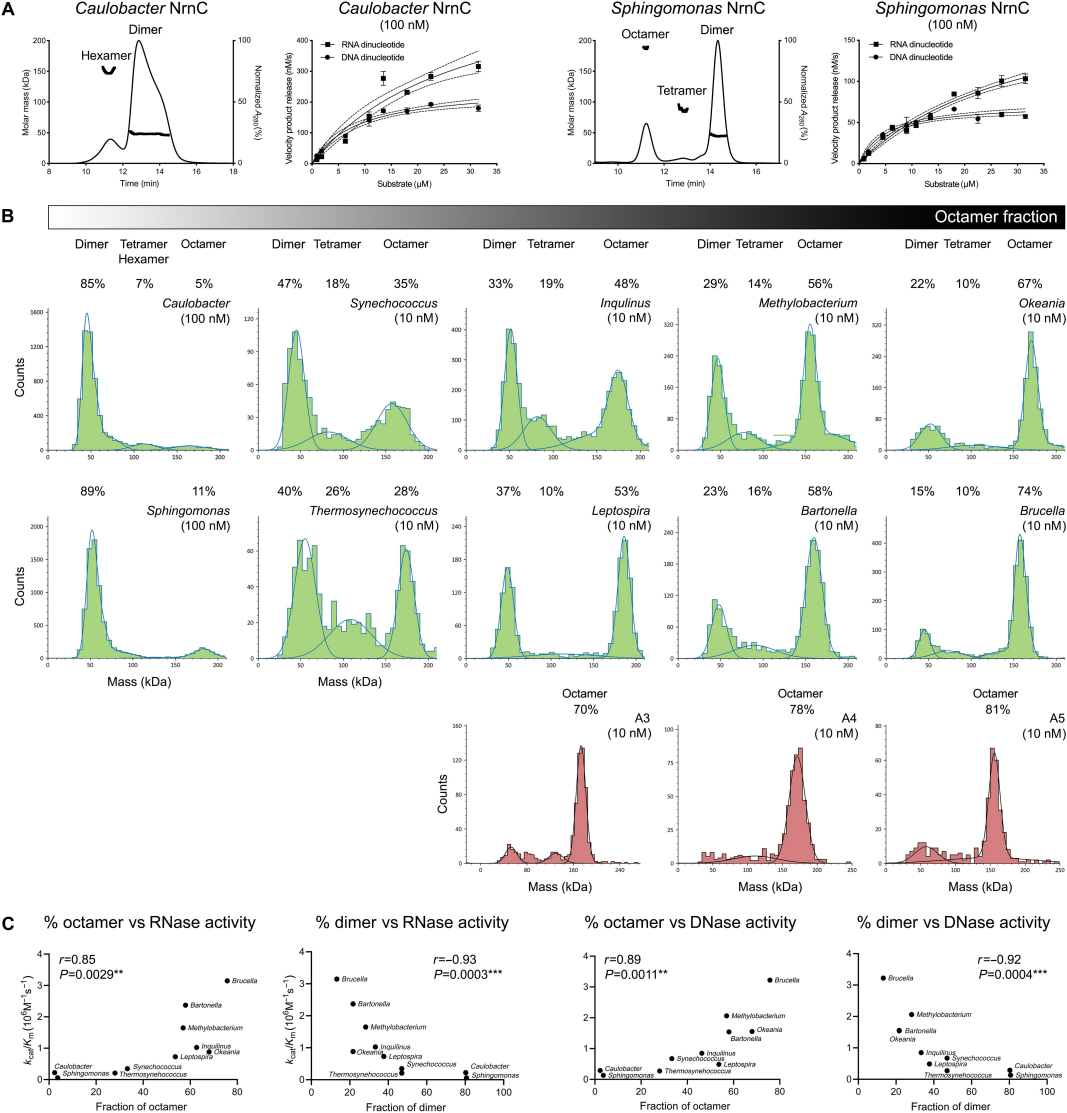
Assessment of NrnC oligomerization and its relationship with enzymatic activity. (**A**) Oligomeric states and activity of *Caulobacter* and *Sphingomonas* NrnC. Oligomeric states were determined by SEC-MALS at micromolar protein concentrations. The normalized absorbance at 280 nm (chromatogram lines, right axis) and molar mass values (dots across peaks, left axis) were plotted against the SEC elution volume. Enzymatic activities of *Caulobacter* and *Sphingomonas* NrnC toward DNA and RNA dinucleotides were measured at 100 nM when the proteins were >85% dimeric. Dotted lines show 95% confidence intervals of the Michaelis-Menten model fit. *A*_280_, absorbance at 280 nm. (**B**) Mass photometry histograms obtained for different NrnC orthologs and ancestors (A3, A4, and A5) at nanomolar concentrations. The mean values of the Gaussian fits correspond to the theoretical molecular masses of the dimer, tetramer, hexamer, and octamer states of the NrnC orthologs. The measurements were performed in triplicate, and representative histograms are shown. (**C**) Correlation between octamer or dimer fractions of 10 different NrnC orthologs and their enzymatic activity levels. The *r* factors and *P* values derived from Spearman’s nonparametric correlation are indicated.

Because two NrnC specification events, the disappearance of DNA preference and the emergence of octamerization, co-occurred, we next investigated whether these characteristics depend on one another or emerged coincidentally. To answer this question, we attempted to create artificial dimeric NrnC mutants to measure enzyme function when the octameric interface of NrnC is broken; however, these variants could not be expressed recombinantly. As an alternative, we assessed whether there were natural NrnC orthologs that would disassemble into smaller subunits at lower protein concentrations, allowing us to measure the catalytic activity of their subunits. We identified two Alphaproteobacterial NrnC orthologs, from *Caulobacter* and *Sphingomonas*, which displayed instability in octameric arrangements in our SEC-MALS experiments. Both orthologs exist as mixtures of dimers, tetramers, hexamers, and octamers at the micromolar concentrations used for the analysis (Fig. 5A, upper panels), consistent with AF3 predictions indicating octamers as feasible oligomeric assemblies of these orthologs (Fig. 3B). When diluted further to a concentration of 100 nM, they appeared almost entirely as dimers (>85%) according to mass photometry, an experimental method better suited for mass determination at low analyte concentrations (Fig. 5B). We used the ability of these NrnC orthologs to remain dimeric at low concentrations to measure the enzymatic activity of NrnC as a function of concentration and, hence, the protein’s oligomeric state. Both *Caulobacter* and *Sphingomonas* NrnC at 100 nM processed RNA and DNA dinucleotides indiscriminately, similar to all other NrnC orthologs tested so far (Fig. 5A, lower panels, and table S4). This result indicates that the absence of DNA preference does not require the octamerization of NrnC.

Because the octamerization of *Caulobacter* and *Sphingomonas* NrnC noticeably depended on protein concentration, we assessed the oligomeric states of all other NrnC orthologs at the concentrations used previously for determining enzyme kinetics. We found that all NrnC orthologs tested existed as mixtures of dimers, tetramers, and octamers at 10 nM, with the fraction of octamers varying across the different proteins (Fig. 5B). From this group, the *Brucella* and *Okeania* NrnC orthologs displayed the highest fraction of octamers. Next, we investigated whether there is a link between the level of octamerization and enzyme activity among NrnC orthologs. To this end, we performed correlation tests using the catalytic efficiency, *k*_cat_/*K*_M_ (catalytic constant over Michaelis constant), values of 10 NrnC orthologs toward either RNA or DNA dinucleotides and their dimer or octamer fractions (Fig. 5, B and C; fig. S3, A and B; and table S4). We found that for both substrates, there was a strong positive correlation between enzyme activity and octamer fraction (Spearman’s nonparametric correlation *r* factors of 0.85 and 0.89 and *P* values of 0.0029** and 0.0011** for RNase and DNase activity, respectively) and a strong negative correlation between the level of activity and dimer fraction (Spearman’s nonparametric correlation *r* factors of −0.93 and −0.92 and *P* values of 0.0003*** and 0.0004*** for RNase and DNase activity, respectively) (Fig. 5C). Moreover, for the enzyme with the highest octamer fraction, NrnC from *Brucella*, we observed enzyme kinetics that fit a sigmoidal curve, indicating a degree of positive cooperativity (fig. S3, A and B).

The structural analysis revealed that the α4-α5 loop is not only involved in interdimer interactions within octameric assemblies but also takes part in coordinating the 3′ base of the dinucleotide substrate (Fig. 4B), which may enhance substrate binding during catalysis. In this context, we investigated whether the presence of substrates promotes or stabilizes octameric assemblies. To answer this, we measured the oligomeric state distributions of the NrnC orthologs in the presence of a substrate, dpGG (fig. S3C). Some NrnC orthologs, those from *Inquilinus*, *Leptospira*, and *Synechococcus*, showed an increase in octamer population when the substrate was present (Fig. 5B and fig. S3C). However, this phenomenon was not a universal feature, with other orthologs not showing this effect. Accordingly, levels of octameric fractions recorded in the presence of a substrate are again in strong positive correlation with nuclease catalytic efficiencies of different NrnC orthologs (fig. S3D). In summary, these data indicate that octamerization of NrnC orthologs increases global enzymatic activity without affecting substrate specificity.

The correlation between the stability of octameric assemblies in dilute conditions and nuclease activity in extant enzymes prompted us to assess the oligomer stability of NrnC ancestors A3, A4, and A5. These ancestors displayed a high degree of octamerization at 10 nM (Fig. 5B), suggesting that the stability of this quaternary structure was high early in NrnC evolution.

**Fig. 6.**
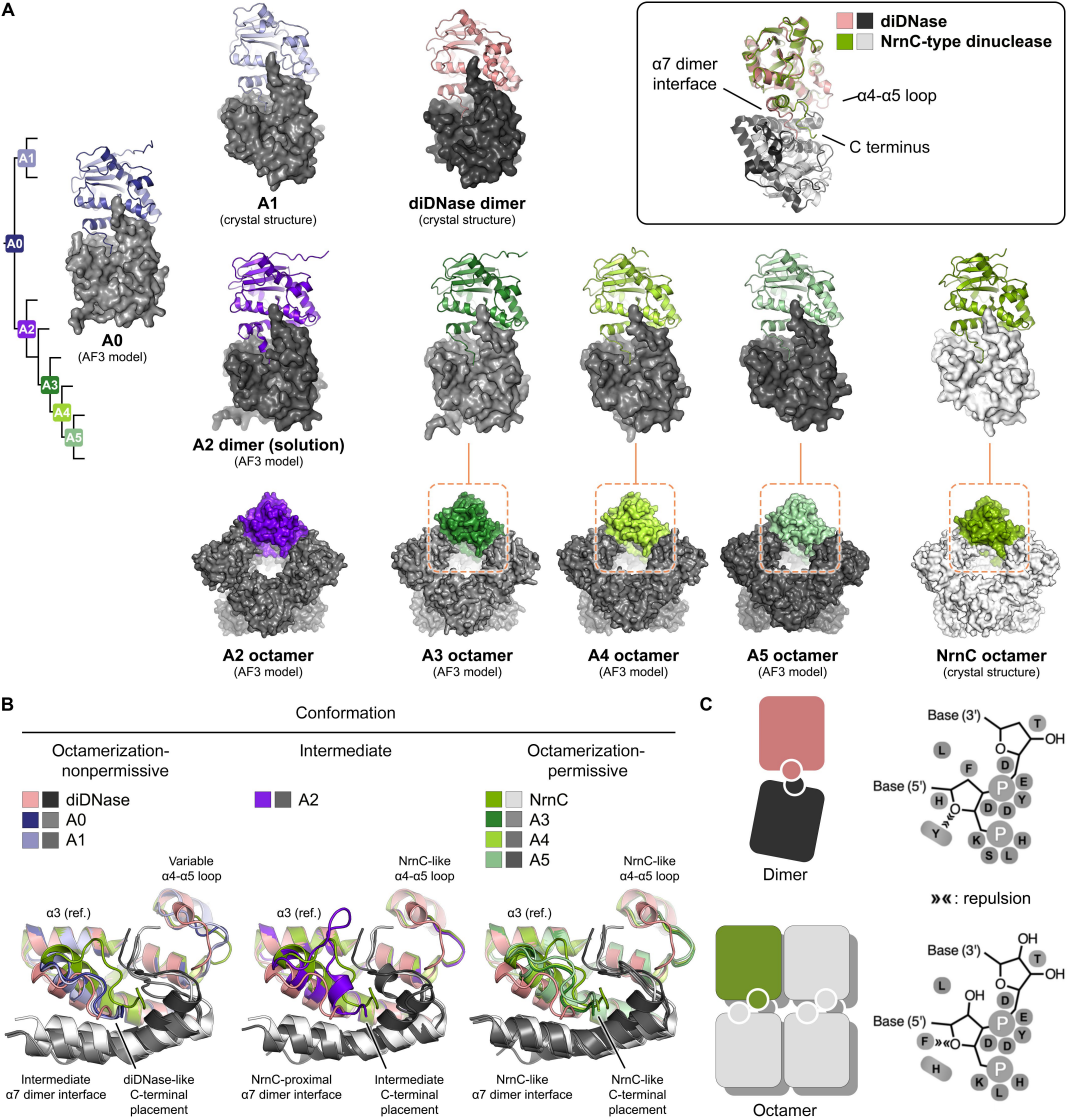
Structural modeling of NrnC and diDNase ancestors. (**A**) AF3 models of ancestor of NrnC and diDNases. Oligomeric states were predicted on the basis of confidence scores from modeling different assemblies (table S6). Dimers or corresponding dimeric subunits extracted from an octamer are shown, with one chain in color and the other in gray. The inset shows a superposition of extant NrnC*_Bh_* and diDNase*_Noc_* using the catalytic motifs of one monomer as a reference. (**B**) Analysis of the isolated dimer interfaces. Dimers of ancestors and extant enzymes were superimposed as described in (A). On the basis of the structural analysis and oligomerization in solution, the interdimer conformations were categorized as octamerization-nonpermissive, intermediate, and octamerization-permissive. (**C**) Model for correlated oligomerization and active-site composition. The dimer interface (i.e., α7 helix dimer) between nuclease subunits determines its oligomeric propensity. The placement of structural elements such as the α4-α5 loop and the C terminus reaching across the dimer interface into an adjacent subunit constrains the active site architecture.

Consistent with their oligomerization characteristics in solution, structure prediction indicates ancestor A0 as a dimer, whereas ancestors A3, A4, and A5 can be modeled as octameric assemblies with high confidence, which resemble octamers determined for extant NrnC orthologs (Fig. 6A and table S6). The superposition of the ancestor models on the structures of an extant NrnC dinuclease (NrnC*_Bh_*) and a diDNase (diDNase*_Noc_*) using the catalytic motifs from one monomer as the reference implicates the α4-α5 loop and the α7 dimer interface in defining the quaternary structure of the ancestors (Fig. 6B). Considering the dimer interface, ancestor models A0 and A1 show more variability in the conformation of the α4-α5 loop, consistent with this loop not being engaged in higher-order oligomerization. The α7 helix dimer at the intermolecular interface adopts a conformation intermediate to that of NrnC and diDNase (Fig. 6, A and B). However, the placement of the C terminus across the dimer interface resembles more that of diDNases. Ancestors A3, A4, and A5 show overall better agreement in these features with the structure of NrnC, adopting an octamerization-permissive conformation. The models are consistent with the propensity of these ancestors to form octamers in solution (Figs. 3C and 5B). The modeling depicts ancestor A2 as a distinct intermediate state (Fig. 6, A and B). Its α4-α5 loop and α7 helix dimer adopt NrnC-like conformations, correlating with the ability of this ancestor to be modeled as an octamer (table S6). The C-terminal placement is in between the placements seen in diDNase and NrnC. However, in solution, ancestor A2 forms constitutive dimers, suggesting an incomplete transition preventing octamer assembly.

## DISCUSSION

The evolution of diDNases and NrnC orthologs provides intriguing insights into the adaptive dynamics of nucleases within shared structural folds. Both enzymes evolved independently from an ancient common ancestor with an intermediate substrate preference. In addition, our findings indicate that mobile diDNases have persisted as distinct enzymes in Actinomycetes and Clostridia, alongside housekeeping NrnC and Orn orthologs. This suggests beneficial roles of diDNase activity in bacterial populations, potentially associated with phage defense or DNA mobility pathways (*8*). Our results also indicate that diDNases have tolerated a relatively high degree of sequence variation, likely driven by their faster evolution rate as part of MGEs (*18*), without affecting the conservation of their substrate preference and oligomeric state. The hallmarks of gene mobility in the entire diDNase phylogenetic clade, such as heterogenic phylum distribution and high sequence diversity, together with the fact that diDNases split from NrnC orthologs early in evolution, suggest that diDNases became mobile early on. It is not possible to determine whether the common diDNase/NrnC ancestor A0 was a mobile gene that became domesticated in the NrnC lineage or whether the ancestor was a core gene that was mobilized in the diDNase branch. Because of the close coevolution of organisms and MGEs, both scenarios are conceivable (*19*). In addition, whether the ancestor A0 itself derived from an even more promiscuous general nuclease remains an open question that would require reconstructing deeper ancestral nodes.

Both diDNases and NrnC enzymes appear to have evolved from a low-activity ancestor and progressively developed higher enzymatic activity, with diDNases specializing in DNA and NrnC orthologs developing a broader substrate preference. This suggests that the primary evolutionary driver for both groups was the advantage of high nuclease activity levels. Simultaneously, NrnC-specific evolutionary pressure dictated the maintenance of both RNase and DNase activities at more or less equal levels. This result emphasizes the potential importance of dinuclease activity toward DNA for the biological function of NrnC (and Orn) orthologs, making diDNases dispensable under normal growth conditions. Notably, while both bacterial housekeeping dinucleases, NrnC (*7*, *8*) and Orn (*8*, *20*), and functionally related nanoRNases NrnA (*2*) and NrnB (*3*) show considerable DNase activity, the physiological relevance of this activity has not been explored in detail. Organisms that contain a diDNase gene also encode an Orn ortholog with activity against both DNA and RNA (*8*), suggesting that both enzymes play a role in cellular homeostasis or survival, with diDNases likely providing a more specific, potentially context-dependent function. At the same time, expression of diDNases in a *P. aeruginosa orn* deletion strain yielded partial complementation, indicating that the activity of Orn orthologs against DNA dinucleotides likely contributes to the full spectrum of the deletion phenotype (*8*). In this regard, the ancestors of diDNases and NrnC orthologs resemble diDNases, providing only a partial rescue of the *orn* deletion, tracking their substrate preferences in enzyme assays (Fig. 1, B and C). This result also highlights the physiological importance of increased RNase activity in the Orn and NrnC clades, an activity that is crucial for full complementation of *orn* deletion (*4*, *7*, *8*, *21*).

We showed that the propensity of NrnC orthologs to form octamers correlates with higher catalytic efficiency, suggesting that higher-order assembly may contribute to enhanced enzymatic activity. The cooperativity of enzymatic catalysis and ligand binding driven by protein self-assembly is a common phenomenon readily explained by simple geometrical considerations and protein conformation flexibility (*22*–*24*). Homomeric proteins with dihedral symmetries, such as the tetramer of dimers of NrnC, were shown to be particularly enriched in allosteric properties (*25*). However, NrnC octamerization does not appear to be strictly required for the processing of RNA and DNA dinucleotides, given that some NrnC orthologs disassemble into dimers under dilute conditions and still cleave both types of substrates equally well (Fig. 5). This observation does not rule out the possibility that octamerization may have been important in the emergence of the promiscuous substrate profile during evolution, especially considering that ancestral proteins of the NrnC lineage form rather stable octamers even in dilute conditions (Fig. 5B). The stability of octamers may have become less crucial for the physiological function later in evolution, possibly leading to the observed variability of octamerization propensity among the extant, fully specialized NrnC proteins. On the basis of our structural analysis, octamerization requires a certain dimer subunit configuration (Figs. 4 and 6, A and B, and fig. S2C); however, we also observed that this configuration and its associated localized conformational changes play a crucial role in widening the active site, allowing RNA dinucleotides to bind (Fig. 2) (*8*). In contrast, the arrangement of protomers within diDNase dimers is incompatible with octamerization, and the active sites are optimized for DNA dinucleotide binding (Figs. 2 and 6C and fig. S2C). Hence, it is conceivable that octamerization aided in the rearrangement at the dimer interfaces, rendering NrnC orthologs as robust enzymes that maintain high catalytic efficiency toward both RNA and DNA substrates. At low enzyme concentrations, the octamers break apart, but the particular dimer subunit configuration persists, explaining the unaltered substrate specificity. Losing contacts at the interdimer interface with the 3′ base of the substrate upon octamer disassembly may explain the reduced overall activity under these conditions (Figs. 4 and 5).

Octamerization of NrnC relies on heterologous interfaces between the dimeric building blocks, which involve main-chain interactions as well as a minimal set of side chains that form a salt bridge, hydrogen bonds, and a cation-π interaction. The interface appears weak on its own, but its stability is likely increased by the geometric constraints dictated by the octameric assembly, in which the interface occurs four times when considering the tetramerization of dimeric subunits. The evolutionary emergence of the interfaces and shape complementarity between them required many substitutions according to our structural analysis and was not achieved in a discrete evolutionary step on the basis of our ancestral reconstruction. This trajectory is similar to the acquisition of the cooperative tetrameric form of vertebrate hemoglobin (*26*, *27*). In hemoglobin, the residues of the interacting interfaces were fixed first, setting the context for the second step: the substitution of just one residue that allowed the higher-order oligomer to form using the interfaces acquired in the first step. In NrnC, we did not observe such discrete steps when analyzing the ancestral and extant enzymes (Fig. 3). However, it is clear that once the octamer arose, it was maintained as an essential feature of the NrnC clade required for high enzymatic activity and, probably, protein stability.

Some recent experimental reports on protein oligomerization have argued that the formation of higher oligomers in metabolic enzymes or other proteins is generally a neutral evolutionary event (*14*, *28*–*32*). Enzymes performing analogous functions to NrnC in other bacteria, namely Orn, NrnA, and NrnB, are dimeric and do not form higher assemblies; however, their nuclease activity is comparable to that of extant NrnC orthologs (*2*–*4*, *8*). Likewise, diDNases, which are structurally related to NrnC, gained higher catalytic efficiency compared to their ancestors but only form constitutive dimers. Hence, although high dinuclease activity does not require larger oligomeric assemblies per se, octamerization yielded one way that correlates with increased enzyme activity, a widened substrate preference, and potentially other functional implications. These could include enabling or preventing interactions with other cellular factors, introducing allosteric regulation or cooperativity, or serving as an additional size filter, considering that the active sites line the walls of a narrow channel formed by the octamers.

## MATERIALS AND METHODS

### Phylogenetic and ancestral sequence reconstructions

Protein sequences from the representative genomes of the Genome Taxonomy Database (release 212) (*33*) were annotated using eggNOG-mapper (version 2.1.3) (*34*) and InterProScan (version 5.64-96.0) (*35*). Sequences annotated as belonging to COG0349 (ribonuclease D) or having an annotated PF01612 domain (3′-5′ exonuclease) were selected and clustered at 60% identity using CD-HIT (version 4.8.1) (*36*). The sequence dataset was curated to remove exceptionally long or short sequences (less than 150 amino acids or greater than 400 amino acids) on the basis of the lengths of the known NrnC and diDNase proteins. The remaining sequences were supplemented with 44 sequences from experimentally characterized NrnC and diDNase proteins, yielding a total dataset of 2401 NrnC, diDNase, and related nucleases.

The sequences were aligned using MAFFT (version 7.475) (*37*), and the alignment was trimmed using the “gappyout” method in trimAL (version 1.5.0) (*38*). A phylogenetic tree was constructed using IQ-TREE (version 2.3.5) (*39*) with the best-fit model (LG amino acid exchange rate matrix, empirical amino acid frequencies, with the FreeRate model of rate heterogeneity) and 10,000 bootstraps. The phylogenetic tree was manually rooted on the basis of the positioning of the experimentally characterized NrnC and diDNase proteins to separate the clades of interest from other related nuclease sequences. The ancestral sequence reconstruction was performed on the basis of this reconstructed tree with GRASP with default settings (version 1.0) (*40*). The phylogenetic reconstruction was visualized using iTOL (*41*). The full marginal posterior probability distributions at all reconstructed positions for nodes A0 to A5 are provided in table S7.

### Protein expression and purification

Genes encoding ancestral proteins, codon optimized for expression in *Escherichia coli*, were synthesized and fused with a His_6_-tagged small ubiquitin-like modifier (SUMO) at the N terminus in a modified pET28a vector (GenScript, Novagen). A single-residue substitution in ancestor A2 was introduced using the QuikChange II XL Site-Directed Mutagenesis Kit (Agilent). The resulting constructs were transformed into One Shot BL21(DE3) cells (Invitrogen). Transformed cells were grown in lysogeny broth (LB) supplemented with kanamycin (50 μg/ml) overnight at 37°C with shaking. Autoinduction media ZYM-5052 (*42*) supplemented with kanamycin (100 μg/ml) was inoculated at a 1/100 ratio with the overnight culture, grown at 37°C with shaking for 2 hours, and then grown at 18°C with shaking for 18 to 20 hours. Cells were harvested by centrifugation, and the pellets were stored at −70°C until purification.

For purification, cell pellets were thawed and resuspended in lysis buffer [50 mM tris-HCl, 500 mM NaCl, 25 mM imidazole, 1 mM phenylmethylsulfonyl fluoride, and 3 mM β-mercaptoethanol (pH 8.5)]. Cells were lysed by sonication, and the lysates were clarified from insoluble material by centrifugation. Clarified lysates were incubated with Ni-NTA resin (Qiagen) under gentle agitation at 8°C for 1 hour. The resin was collected by centrifugation, washed three times with five volumes of lysis buffer, and placed in a gravity column. His_6_-tagged Ulp1 protease was added to the Ni-NTA resin in 1 to 2 resin volumes of lysis buffer, the column was sealed, and the resin was incubated at 8°C for 1 hour with rotation to allow for cleavage of the His_6_-SUMO tag. Subsequently, the flow-through containing the untagged protein of interest was collected and concentrated using centrifugal filter units (Amicon Ultra-4, 10-kDa cutoff). EDTA (pH 8.5, final concentration of 10 mM) was added to the concentrated protein to chelate divalent cations and further purified by gel filtration using either HiLoad 16/600 Superdex 200 pg or Superdex 200 Increase 10/300 GL columns (Cytiva) equilibrated with gel-filtration buffer [for ancestor A0, 25 mM Hepes-NaOH and 500 mM NaCl (pH 7.5); for all others, 25 mM Hepes-NaOH and 150 mM NaCl (pH 7.5)]. Protein-containing fractions were pooled, concentrated, flash-frozen in liquid nitrogen, and stored at −70°C.

### Size-exclusion chromatography–coupled multiangle light scattering (SEC-MALS)

Purified proteins at 1 to 2 mg/ml (∼40 to 80 μM) were injected onto a Superdex 200 Increase 10/300 GL column (Cytiva) equilibrated with gel-filtration buffer at room temperature. The gel-filtration setup was coupled inline to a static multiangle light scattering detector (miniDAWN, Wyatt Technology, Waters) and a refractive index detector (Optilab T-rEX, Wyatt Technology, Waters). Data analysis was performed using Astra software (version 8.0.2.5, Wyatt Technology, Waters), yielding molar mass values across the elution peaks of the samples, as well as the weight fractions of each molar mass species.

### Enzyme kinetics

Substrate hydrolysis by NrnC homologs was measured by following the fluorescence unquenching of nucleotides containing 2-aminopurine as one of the bases over time (*43*). All substrates were purchased 5′-phosphorylated from the manufacturer (Dharmacon). Enzymatic activity was measured as the rate of increase in the fluorescence intensity of cleaved 2-aminopurine, with excitation at 310 nm and emission at 375 nm, over a series of initial substrate concentrations (0.9 to 45 μM). Enzyme concentrations ranged from 10 to 100 nM, depending on the level of activity, and are indicated in the results. The reactions were assembled by mixing the reaction buffer [20 mM tris-HCl and 150 mM KCl (pH 7.9)] with a substrate stock prepared in water and an enzyme diluted with dilution buffer [20 mM tris-HCl, 150 mM KCl, and 4 mM EDTA (pH 7.9)]. The resulting mixtures (45 μl) were placed in wells of a 96-well plate, and the reactions were initiated by adding 5 μl of start buffer [20 mM tris-HCl, 150 mM KCl, and 100 mM MgCl_2_ (pH 7.9)]. The fluorescence intensity was recorded in triplicate every 10 s for 3 min at 21°C using a Tecan Infinite M Plex plate reader. The initial slopes of the resulting time-course data were determined and converted into product release velocities using a calibration curve. Calibration curves were created for each type of substrate tested by incubating the reactions for 5 to 10 hours in a sealed plate to allow for complete substrate conversion and by plotting the final fluorescence intensity values against the initial substrate concentrations. The velocities of product release were plotted against the initial substrate concentrations and fitted with enzyme kinetics models in GraphPad Prism 10.

### Crystallization and determination of structures

All crystals were obtained in sitting drops at 19°C. *Okeania* NrnC-pGG was crystallized in 0.2 M lithium chloride and 20% polyethylene glycol, molecular weight 3350 (PEG-3350). *Inquilinus* NrnC was crystallized in 0.2 M magnesium chloride, 0.1 M bis-tris (pH 5.5), and 25% PEG-3350. Ancestor A1 was crystallized in 0.1 M MES (pH 5) and 20% (w/v) PEG-6000. The crystals were cryoprotected using either xylitol or low-viscosity oil before flash cooling in liquid nitrogen. Diffraction data were collected at beamline P11 at the Deutsches Elektronen-Synchrotron (DESY) and processed using XDS (*44*). The phase information was obtained by molecular replacement using Phaser (*45*). The models were iteratively refined using Phenix.refine (*46*) and manually built using Coot (*47*). Structural figures were prepared using PyMOL (version 2.5.0; Schrödinger LLC). The data processing and model refinement statistics are presented in table S5.

### *P. aeruginosa* Δ*orn* complementation assay

Genes of interest were subcloned into a modified pJN105 vector (*48*) to express N-terminal StrepII-tagged proteins in *P. aeruginosa* Δ*orn*. The plasmids were introduced into *P. aeruginosa* via electroporation. Briefly, cells from an overnight culture grown in LB were collected and washed three times with 1 mM MgCl_2_, followed by the addition of plasmid DNA and electroporation of the cells. Cells were recovered in 1 ml of LB with shaking at 250 rpm for 1 to 2 hours at 37°C and plated on LB agar containing gentamicin (60 μg/ml). For complementation assays, fresh LB supplemented with gentamicin (60 μg/ml) was inoculated with cells harboring a plasmid, followed by overnight growth. The resulting cultures were diluted to an optical density at 600 nm of 0.1, followed by preparation of a serial dilution series. Each dilution (12 μl) was applied to LB agar plates containing gentamicin (60 μg/ml) and 0.2% arabinose. The plates were inverted, allowing the culture to drip down the length of each plate. The plates were incubated overnight at 37°C. The plates were imaged, and the colony areas of well-separated colonies were determined using particle analysis in ImageJ (*49*). The area of each colony was divided by the average area of wild-type *P. aeruginosa* colonies grown on the same plate to allow normalization across all plates. A minimum of 50 colonies was quantified.

### Western blotting

*P. aeruginosa* expressing StrepII-tagged ancestors from a plasmid was cultured in LB supplemented with gentamicin (60 μg/ml) and 0.2% arabinose overnight at 37°C. Cells (1 to 3 ml) from each culture were collected and resuspended in SDS–polyacrylamide gel electrophoresis loading buffer. The samples were incubated at 100°C for 10 min and resolved on 15% SDS–polyacrylamide gel electrophoresis gels. Proteins were transferred onto a 0.2-μm polyvinylidene difluoride membrane using a Trans-Blot Turbo Transfer System (Bio-Rad). The membranes were blocked with EveryBlot Blocking Buffer (Bio-Rad) for 1 hour at 20°C, followed by incubation with StrepTag II Antibody-HRP Conjugates (Millipore, Merck, at a 1:10,000 dilution) for 1 hour at 20°C. Membranes were washed three times for 5 min with phosphate-buffered saline with 0.05% Tween 20 and imaged with Amersham ECL Select Western Blotting Detection Reagent (Cytiva) for chemiluminescence detection.

### Mass photometry

Diluted protein (12 μl, 20 to 200 nM) was mixed with an equal volume of phosphate-buffered saline directly before the measurement. For measurements in the presence of a substrate, 1 μM dpGG was added to the samples and buffer. Data were recorded at 100 frames per second for 60 s using AcquireMP (Refeyn Ltd., version 1.2.1) on a TwoMP instrument (Refeyn Ltd.) and analyzed using DiscoverMP (Refeyn Ltd., version 2022 R1). Each protein was measured at least thrice.

### AF3 modeling of oligomeric states

The oligomeric structures were modeled using AF3 (*50*) on the AlphaFold Server with default settings. ipTM scores of extant orthologs are reported in Fig. 3B. Modeling scores for the ancestors are provided in table S6.
