# Supplementary material for: Ancestral proteins trace the emergence of substrate specificity and oligomerization within bacterial DEDDy dinucleases
Source: Sci Adv. 2026 Aug 12;12(33):eaef1581. doi: 10.1126/sciadv.aef1581 (PMC13464484; doi:10.1126/sciadv.aef1581)
Supplement: Supplementary file 1 [file sciadv.aef1581_sm.pdf]

Science Advances
AAAS

Supplementary Materials for

Ancestral proteins trace the emergence of substrate specificity and oligomerization within bacterial DEDDy dinucleases

Sofia Mortensen *et al.*

Corresponding author: Sofia Mortensen, sofia.mortensen@desy.de; Holger Sondermann, holger.sondermann@cssb-hamburg.de

Sci. Adv. **12**, eaef1581 (2026)
DOI: 10.1126/sciadv.aef1581

The PDF file includes:

Figs. S1 to S3
Tables S1 to S6

Other Supplementary Material for this manuscript includes the following:

Table S7

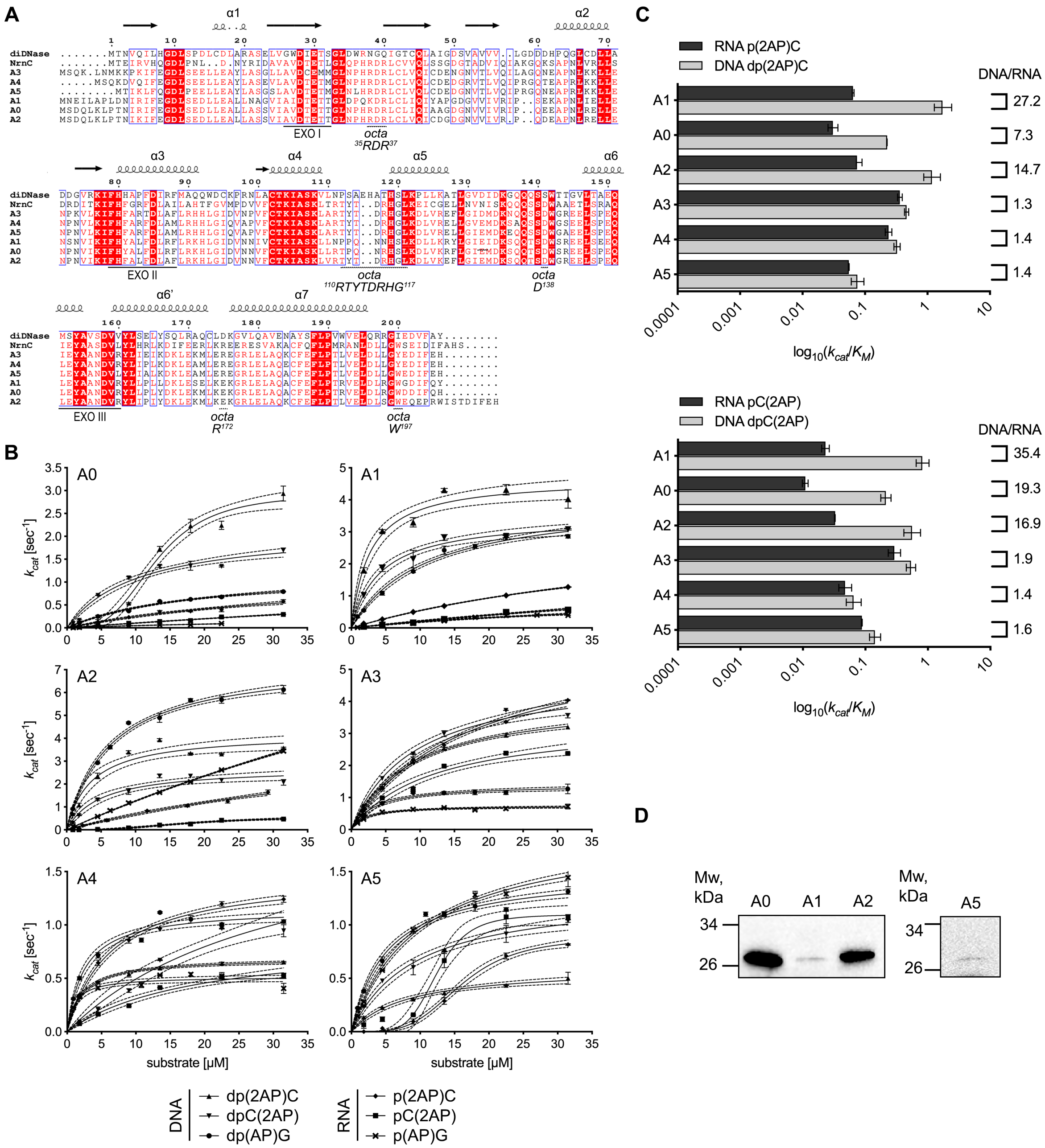

Fig. S1. Sequence and functional analyses of ancestral proteins. A. Sequence alignment of ancestor proteins at nodes of interest and *Bartonella henselae* NrnC (NrnC$_{Bh}$) and *Nocardioides* diDNase (diDNase$_{Noc}$). **B**. Enzymatic activity of ancestral proteins towards RNA (p[2AP]G, p[2AP]C, pC[2AP]) and DNA (dp[2AP]G, dp[2AP]C, dpC[2AP]) substrates. The data were fitted using either a Michaelis-Menten or allosteric sigmoidal enzyme kinetics model and plotted as k_{cat} *vs* initial substrate concentration. Dotted lines show 95% confidence intervals of the model fit. **C**. Catalytic efficiency of ancestral proteins toward DNA and RNA dinucleotides used in **B**. The values of k_{cat}/K_M ($\times 10^6$ M^{-1} s^{-1}) are plotted on a $\log_{10}$ scale. The error bars show the 95% confidence interval of the Michaelis-Menten model fitting. **D**. Western blot detection of the indicated StrepII-tagged ancestral proteins expressed from an inducible plasmid in the *P. aeruginosa Δorn* strain. The expression of NrnC$_{Bh}$ and diDNase$_{Noc}$ was established in (8).

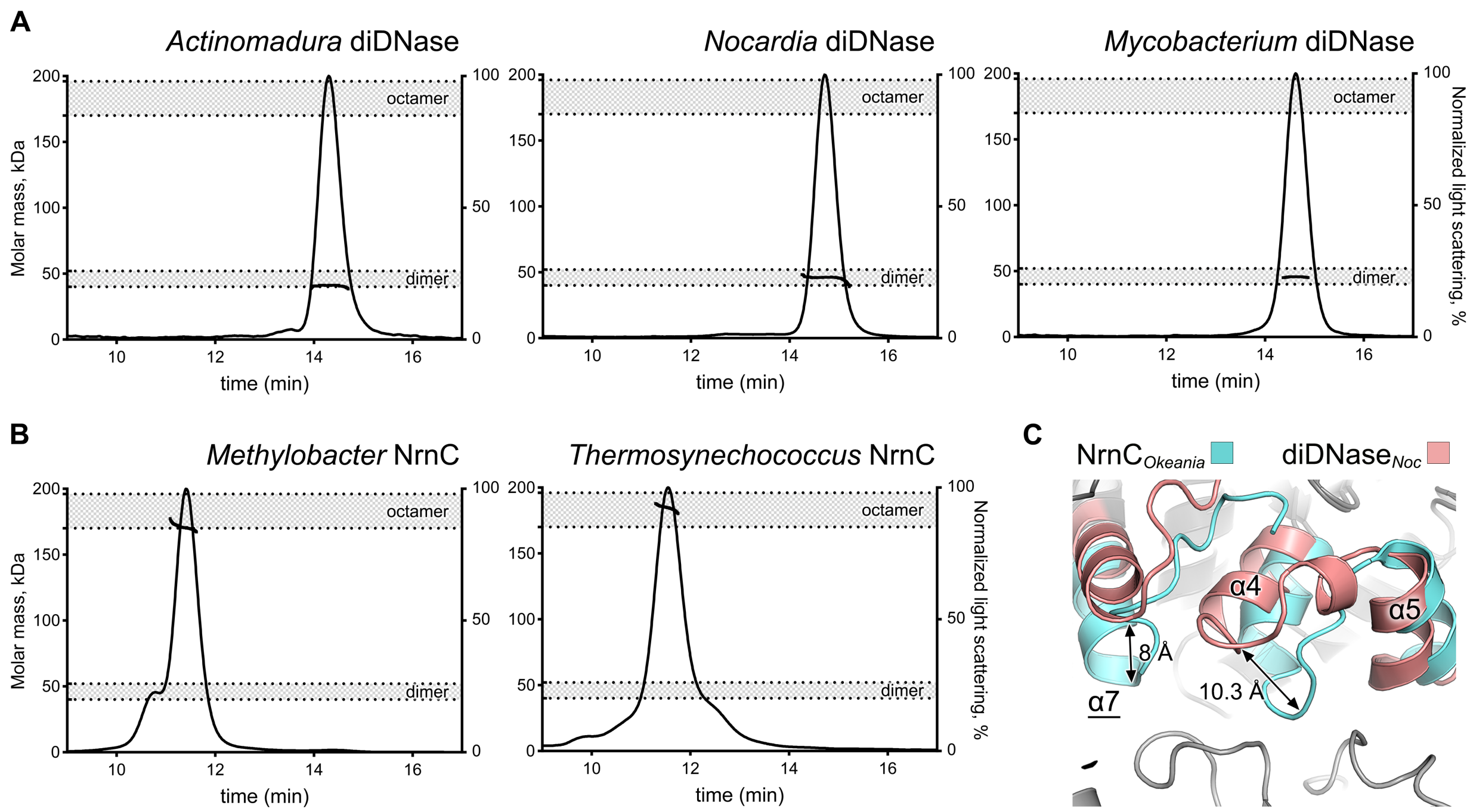

Fig. S2. Oligomeric states of diDNases and NrnC orthologs. A. SEC-MALS analysis of diDNases from *Actinomadura*, *Nocardia*, and *Mycobacterium*. The normalized light scattering signal (chromatogram lines, right axis) and molar mass values (dots across peaks, left axis) were plotted against the SEC elution volume. The shaded areas show the theoretical molar mass ranges for dimeric and octameric assemblies based on the primary sequence of the proteins. **B.** SEC-MALS analysis of *Methylobacter* and *Thermosynechococcus* NrnC. **C**. Close-up view of the NrnC octameric interface area superimposed on a dimeric diDNase structure (PDB ID 9F7G). Major differences in the position of the interacting helices α4, α5 and α7, and loop α4-α5 are indicated by arrows and displacement measurements. Labels for secondary structure elements of protomer B are underlined.

A

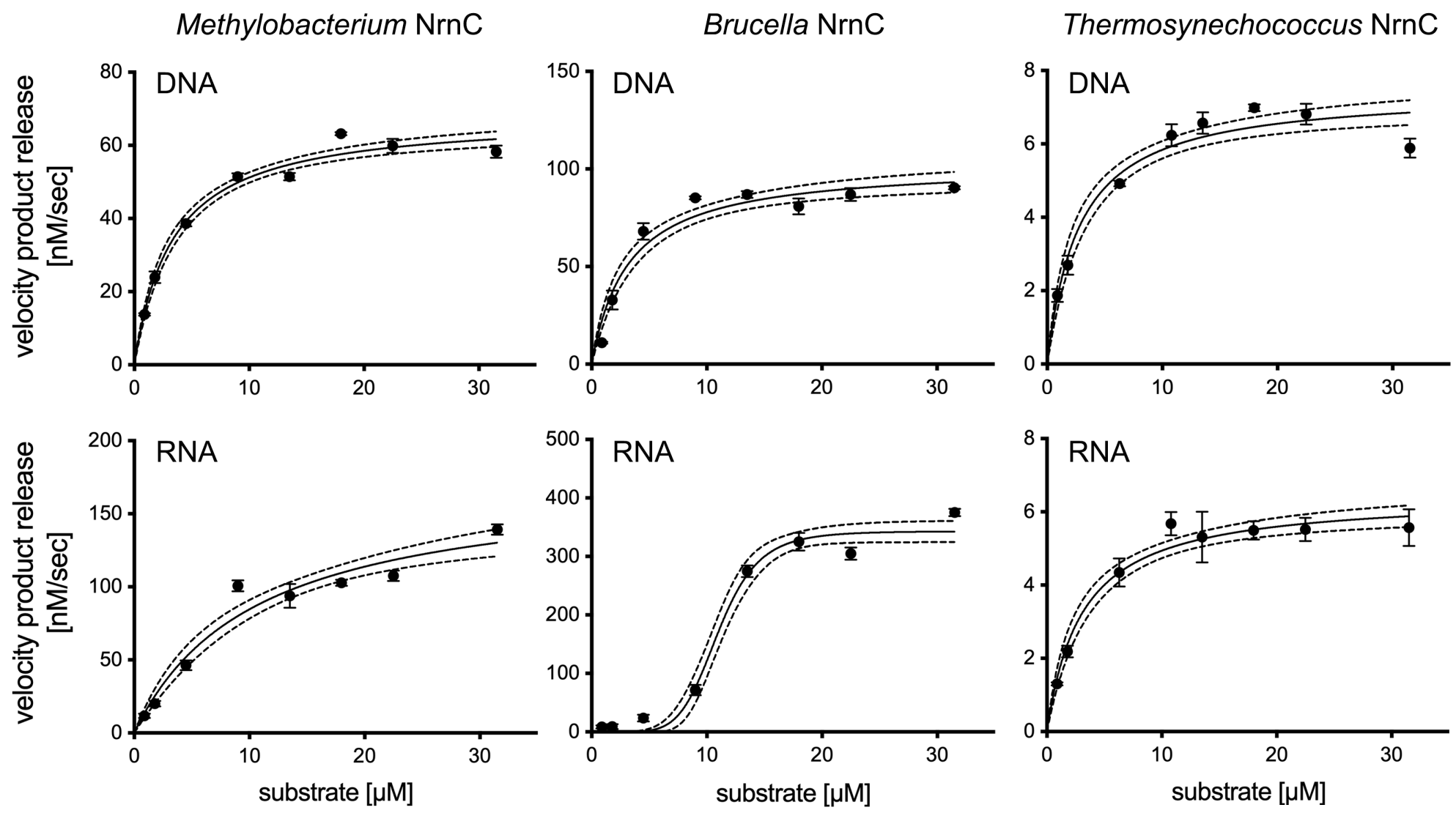

B

	Sphingomonas		Bartonella*		Inquilinus*		Synechococcus*		Okeania*	
	RNA	DNA	RNA	DNA	RNA	DNA	RNA	DNA	RNA	DNA
k_{cat}/K_M, *10^6 $M^{-1}sec^{-1}$	0.06	0.13	2.37	1.54	1.02	0.85	0.35	0.67	0.88	1.56
Hill slope			2.3							
	Leptospira*		Caulobacter		Thermosyn.		Brucella		Methylobacterium	
	RNA	DNA	RNA	DNA	RNA	DNA	RNA	DNA	RNA	DNA
k_{cat}/K_M, *10^6 $M^{-1}sec^{-1}$	0.73	0.49	0.22	0.29	0.21	0.27	3.12	3.22	1.65	2.06
Hill slope							6.3			

* from (8)

C

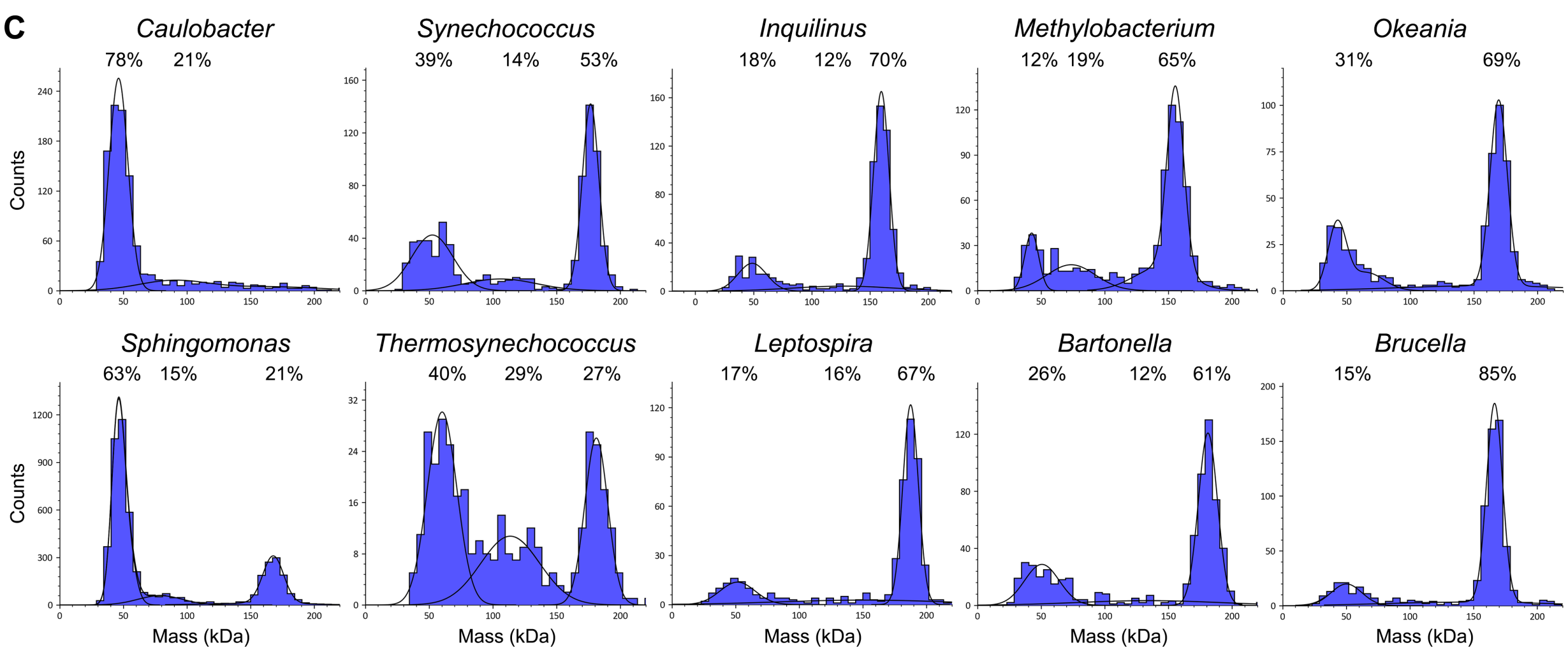

D

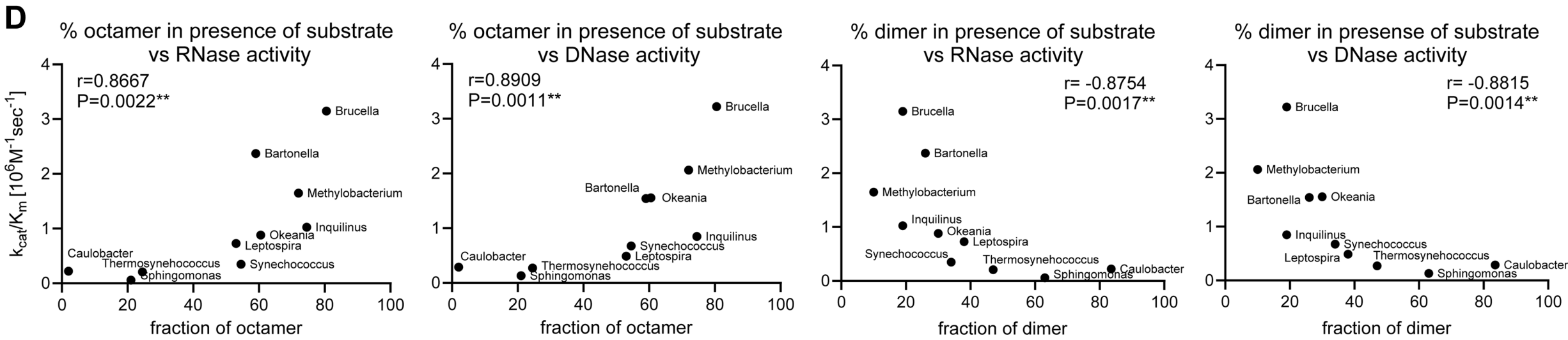

Fig. S3 Kinetic parameter and octamerization of NrnC. A. Enzymatic activities of three NrnC orthologs towards DNA and RNA dinucleotides. Data points were measured in triplicate and fitted using Michaelis–Menten or allosteric sigmoidal models. Mean values and standard deviations (SDs) were plotted. The dotted lines indicate the 95% confidence intervals of the model fitting. **B**.

Catalytic efficiencies, k_{cat}/K_M, of ten different NrnC orthologs towards RNA and DNA dinucleotides. The catalytic efficiency values were used for the correlation analysis shown in Figure 5C. **C**. Representative mass photometry histograms of NrnC orthologs measured at 10 nM protein concentration in the presence of 1 µM dpGG. Fractions of dimeric (left), tetrameric (middle), and octameric (right) assemblies are indicated as percentages. **D**. Correlation between octamer or dimer fractions of 10 different NrnC orthologs recorded in the presence of dpGG substrate and their enzymatic activity levels. The r factors and P values derived from Spearman's nonparametric correlation are indicated.

Table S1.

Table S1. Enzyme kinetic parameters of the ancestral proteins towards DNA and RNA dinucleotide substrates with sequence (2AP)G.

	A0		A1		A2		A3		A4		A5	
	DNA	RNA	DNA	RNA	DNA	RNA	DNA	RNA	DNA	RNA	DNA	RNA
[E], nM	100	100	10	100	10	10	10	10	10	10	10	10
V_{max}, nM/sec	126.4	27.3	40.5	108.0	74.2	178.0	14.1	7.5	11.6	5.2	15.0	19.1
K_M, µM	18.7	46.7	10.8	47.3	6.4	131.6	3.4	2.1	2.5	1.1	5.2	9.8
k_{cat}, s^{-1}	1.26	0.27	4.05	1.08	7.42	17.80	1.41	0.75	1.16	0.52	1.50	1.91
k_{cat}/K_M, $*10^6$ $M^{-1}s^{-1}$	0.068	0.006	0.375	0.023	1.169	0.135	0.418	0.351	0.468	0.475	0.292	0.195
DNA/RNA	11.33		16.30		8.66		1.19		0.99		1.50	

Table S2.

Table S2. Enzyme kinetic parameters of the ancestral proteins towards DNA and RNA dinucleotide substrates with sequence (2AP)C.

	A0		A1		A2		A3		A4		A5	
	DNA	RNA	DNA	RNA	DNA	RNA	DNA	RNA	DNA	RNA	DNA	RNA
[E], nM	100	100	10	100	10	10	10	10	10	10	10	10
V_{max}, nM/sec	293.2	127.8	46.8	342.4	42.2	43.0	41.3	60.5	6.9	14.8	6.0	8.8
K_M, µM	13.0	41.8	2.7	53.2	3.6	57.8	8.9	16.7	2.1	6.2	8.0	16.0
k_{cat}, s^{-1}	2.93	1.28	4.68	3.42	4.22	4.65	4.13	6.05	0.69	1.48	0.60	0.88
k_{cat}/K_M, $*10^6$ $M^{-1}s^{-1}$	0.226	0.031	1.740	0.064	1.178	0.080	0.463	0.362	0.325	0.238	0.076	0.055
Hill slope	3.3											3.9
DNA/RNA	7.29		27.20		14.73		1.28		1.37		1.38	

Table S3.

Table S3. Enzyme kinetic parameters of the ancestral proteins towards DNA and RNA dinucleotide substrates with sequence C(2AP).

	A0		A1		A2		A3		A4		A5	
	DNA	RNA	DNA	RNA	DNA	RNA	DNA	RNA	DNA	RNA	DNA	RNA
[E], nM	100	100	10	100	10	10	10	10	10	10	10	10
V_{max}, nM/sec	217.3	215.1	34.7	319.1	27.3	5.9	48.7	34.4	20.6	8.8	13.0	11.0
K_M, µM	10.2	196.7	4.3	139.2	4.9	17.9	9.1	11.9	31.7	18.5	9.1	12.4
k_{cat}, s^{-1}	2.17	2.15	3.47	3.19	2.73	0.59	4.87	3.44	2.06	0.88	1.30	1.10
k_{cat}/K_M, $*10^6$ $M^{-1}s^{-1}$	0.212	0.011	0.815	0.023	0.557	0.033	0.536	0.290	0.065	0.047	0.143	0.088
Hill slope						2.6						5.4
DNA/RNA	19.27		35.43		16.88		1.85		1.38		1.63	

Table S4.

Table S4. Enzyme kinetic parameters of NrnC proteins towards DNA and RNA dinucleotide substrates.

	Sphingomonas		*Caulobacter*		*Thermosynechococcus*		*Brucella*		*Methylobacterium*	
	DNA	RNA	DNA	RNA	DNA	RNA	DNA	RNA	DNA	RNA
[E], nM	100		100		10		10		10	
V_{max}, nM/sec	218.3	72.1	629.1	250.5	6.5	7.5	343.1	102.7	173.7	68.2
K_M, µM	34.2	5.4	28.5	8.5	3.1	2.8	11.0	3.2	10.5	3.3
k_{cat}, s^{-1}	2.18	0.72	6.29	2.51	0.65	0.75	34.31	10.27	17.37	6.82
k_{cat}/K_M, $*10^6$ $M^{-1}s^{-1}$	0.060	0.130	0.221	0.294	0.211	0.270	3.116	3.223	1.651	2.055
Hill slope								6.3		
DNA/RNA	0.46		0.75		0.78		0.97		0.80	

Table S5.

Table S5. X-ray diffraction data processing and refinement statistics. *

**Structure**	Ancestor A1	$NrnC_{Okeania}$ • pGG	$NrnC_{Inquilinus}$
**Data collection**			
Resolution range	43.43 - 1.45 (1.50 - 1.45)	41.52 - 3.00 (3.11 - 3.00)	43.14 - 1.75 (1.81 - 1.75)
Space group	C 1 2 1	P 4 2 2	P 4 21 2
Unit cell a, b, c (Å)	70.90 70.95 48.24	103.40 103.40 139.40	120.71 120.71 71.76
Unit cell α, β, ɣ (°)	90 115.81 90	90 90 90	90 90 90
Total reflections	256,038 (25,793)	201,061 (20,544)	994,274 (99,824)
Unique reflections	36,862 (3,622)	7,851 (765)	53,803 (5,247)
Multiplicity	6.9 (7.1)	25.6 (26.9)	18.5 (19.0)
Completeness (%)	96.7 (95.28)	99.4 (100.00)	99.8 (99.54)
Mean I/sigma(I)	14.4 (2.79)	37.2 (5.29)	18.3 (1.66)
R-merge	0.06764 (0.7653)	0.06462 (0.7159)	0.1888 (3.314)
CC1/2	0.998 (0.91)	1.000 (0.956)	0.998 (0.689)
CC*	1.000 (0.976)	1.000 (0.989)	0.999 (0.903)
**Refinement statistics**			
Reflections used in refinement	36,840 (3,617)	7,852 (765)	53,778 (5,240)
Reflections used for R-free	1,600 (157)	788 (75)	862 (84)
R-work	0.1666 (0.2239)	0.2048 (0.4292)	0.1823 (0.3521)
R-free	0.2023 (0.2851)	0.2571 (0.5167)	0.1975 (0.3995)
CC(work)	0.964 (0.938)	0.940 (0.786)	0.958 (0.682)
CC(free)	0.947 (0.893)	0.885 (0.680)	0.979 (0.639)
Number of non-hydrogen atoms	1,802	1,637	3,781
macromolecules	1,649	1,630	3,165
ligands	1	7	6
solvent	152	0	610
Protein residues	202	203	406
RMS(bonds)	0.006	0.010	0.008
RMS(angles)	0.91	1.42	1.01
Ramachandran favored (%)	98.00	88.94	97.51
Ramachandran allowed (%)	2.00	11.06	2.49
Ramachandran outliers (%)	0.00	0.00	0.00
Rotamer outliers (%)	0.54	0.00	0.60
Clashscore	3.01	7.52	4.41
Average B-factor	30.36	106.34	26.34

* Values in parentheses represent outer shell.

Table S6.

Table S6. AF3 confidence scores.

	ipTM	pTM	Average pLDDT
A0 dimer	0.85	0.88	89.60
A1 dimer	0.89	0.91	91.28
A2 dimer	0.80	0.84	86.10
A2 octamer	0.90	0.91	88.29
A3 octamer	0.93	0.94	92.98
A4 octamer	0.93	0.94	92.65
A5 octamer	0.94	0.95	94.07
